# Metabolic profile of goats fed diets containing crude glycerin from biodiesel production

**DOI:** 10.3389/fvets.2023.1236542

**Published:** 2023-08-31

**Authors:** Higor Fábio Carvalho Bezerra, Edson Mauro Santos, Gleidson Giordano Pinto de Carvalho, Juliana Silva de Oliveira, Fabiano Ferreira da Silva, Meiry Rodrigues Cassuce, Ricardo Romão Guerra, Danillo Marte Pereira, Daniele de Jesus Ferreira, Thiago Vinicius Costa Nascimento, Anderson de Moura Zanine

**Affiliations:** ^1^Department of Animal Science, Federal University of Bahia, Salvador, Bahia, Brazil; ^2^Department of Animal Science, Federal University of Paraiba, Areia, Paraíba, Brazil; ^3^Department of Animal Science, State University of Southwest of Bahia, Itapetinga, Bahia, Brazil; ^4^Department of Animal Science, Federal Rural University of Pernambuco, Recife, Pernambuco, Brazil; ^5^Department of Animal Science, Federal University of Maranhão, Chapadinha, Maranhão, Brazil; ^6^Departament of Veterinary Medicine at Sertão, Federal University of Sergipe, Nossa Senhora da Glória, Brazil

**Keywords:** agricultural byproducts, glycerol, histopathology, liver, methanol

## Abstract

Feedlot finishing of goats is a growing practice, but the economic viability of this technology is compromised by the inclusion of ingredients such as corn and soybean. An alternative to minimize this barrier is the use of agroindustry coproducts as substitutes for those ingredients, such as crude glycerol. This study aimed to evaluated the metabolism of crossbred Boer finishing goats fed diets containing crude glycerin from biodiesel production. Thirty-two crossbred, castrated goat of age were distributed in a fully randomized experimental design with four treatments and eight replicates. The experiment lasted 69 days, and goats were fed sorghum silage and concentrate, with the inclusion of crude glycerin in the diet at levels of 0, 50, 100, and 150 g/kg on a dry matter basis. The diets did not have an effect (*p* > 0.05) on the serum urea levels. Increasing dietary crude glycerin levels did not the influence the metabolic or urinary profiles (*p* > 0.05). The liver tissue of the goats fed diets containing the highest crude glycerin inclusion levels showed deleterious effects. The inclusion of crude glycerin with approximately 6.6 g/kg methanol caused deleterious effects to the liver tissue of Boer crossbred goats as the glycerin concentrations increased. However, glycerin levels did not cause deleterious effects on the liver tissue or on the serum or urinary profiles. The use of crude glycerin with lower methanol content is recommended for goat diets.

## 1. Introduction

Feedlot finishing of goats is a growing practice, but the economic viability of this technology is compromised by the inclusion of ingredients such as corn and soybean, which have high acquisition costs for farmers. An alternative to minimize this barrier is the use of agroindustry coproducts as substitutes for those ingredients, such as crude glycerol (a glycerol-rich alternative), a coproduct from the production of biodiesel.

Glycerol is a component of normal animal metabolism found in the bloodstream and in cells and is easily employed by animal bodies (1), where it can be absorbed directly by the ruminal epithelium, metabolized in the liver, directed to gluconeogenesis, and converted into glucose (1–3). Nevertheless, glycerol is not used in the animal feed; instead, glycerin, a product resulting from the biodiesel process and rich in glycerol (1,2,3-propanetriol) and containing other components, such as lipids, salts, water, and methanol, is used (4).

In ruminants, glycerol is completely fermented by ruminal fermentation to volatile fatty acids, especially propionate and butyrate (4–6), which decrease ruminal pH and cause negative effects on ruminal microbial protein synthesis, and ruminal fermentation (7).

According to the Brazilian Ministry of Agriculture, Livestock and Supply (8), the national glycerin standard for animal feeds is at most 150 ppm of methanol and at least 80% glycerol. Therefore, standardization of the process is essential for the use of glycerin as an ingredient in animal feed. However, rules to prohibit the marketing of glycerin that do not meet these requirements are lacking, and inspection of those levels is non-existent. Reports on disorders as hepatic degeneration, intoxic by heavy metals and metabolic disorders caused by glycerol were not found in the literature (9–12), although the results are inconsistent. Concerns exist around the use of glycerol residues in animal feeding.

The inconsistency in results may be due to the glycerol purity, duration of supplementation, the speed with which glycerol is fermented in the rumen, and the absorption of glycerol, which is metabolized in the liver, in the rumen epithelium (13). The inconsistency between experiments reveals that more experiments are recommended to validate or refute the importance of including glycerol as a feed ingredient or feed supplement in the diet of ruminants.

Diets rich in substances such as methanol can cause changes in ruminal physiology and, depending on the type of feed, can affect the microorganism population, feed passage rate, nutrient motility, and absorption speed. These factors can cause a series of metabolic disorders that can lead to animal efficiency production losses, particularly to economic losses to farmers (14, 15).

The evaluation of the metabolic profile of goats submitted to new dietary systems using clinical biochemistry, including the determination of serum concentrations of protein and energy profiles and of enzymes related to liver activity (such as alanine aminotransferase, aspartate aminotransferase, and gamma-glutamyl transferase) because these indicators help diagnose metabolic disorders and other diseases.

In view of the above, this study aimed to assess the effect of the inclusion of crude glycerin in the diet of feedlot-finished goat kids on metabolic, protein, energy, and urinary profiles, as well as to provide a histopathological evaluation of liver tissues.

## 2. Materials and methods

Goats were cared for in accordance with the guidelines for the care and use animals presented in the guide issued by the National Institute of Health and by Brazil’s Ministry of Brazil. Federal University of Bahia Animal Use and Care Committee (n. 08/2013).

### 2.1. Location, animals, and diets

The experiment was conducted at the Experimental Farm of the School of Veterinary Medicine and Animal Science of the Federal University of Bahia, in the municipality of São Gonçalo dos Campos, state of Bahia, Brazil, between November 2013 and January 2014.

Thirty-two Boer crossbred goats with initial weights of 17.8 ± 2.2 kg and ages ranging from three to four months were tested, distributed in a fully randomized design with four treatments and eight replicates. The treatments corresponded to the four levels of crude glycerin (0, 50, 100, and 150 g/kg) on a dry matter (DM) basis (Table 1).

**Table 1:** Composition of ingredients and chemical composition of the experimental diets.

Ingredient	Dietary crude glycerin level (g/kg of DM)			
	0	50	100	150
Dietary ingredient (g/kg)
Corn grounded	180.00	120.00	60.00	0.00
Soybean meal	205.00	215.00	225.00	235.00
Crude glycerin	0.00	50.00	100.00	150.00
Mineral supplement	15.00	15.00	15.00	15.00
Sorghum silage	600.00	600.00	600.00	600.00
Chemical composition (g/kg)
Dry matter	554.60	557.20	559.80	562.30
Organic matter^1^	941.00	937.10	940.80	941.80
Mineral matter^1^	50.80	52.30	53.90	55.40
Crude protein^1^	149.20	149.80	150.50	151.10
Ether extract^1^	31.30	28.40	25.50	22.60
Neutral detergent fiber^1^	349.40	343.10	336.80	330.50
Acid detergent fiber^1^	166.70	166.30	165.90	165.50
Nonfiber carbohydrate^1^	419.30	426.40	433.30	440.40
TDN^2^	639.80	639.90	639.90	640.00
Methanol^1^	0.00	3.30	6.60	9.90

^1^Value expressed in g/Kg of dry matter. ^2^Total digestible nutrients estimated.

Animals were vaccinated and dewormed for ectoparasites and endoparasites in the pre-experimental period; then, they were housed in individual stalls in a covered shed with slatted suspended floors and equipped with drinking fountains and feeding troughs to ensure *ad libitum* access to water and feed.

The animals were housed in individual 2-m^2^ pens in a covered sheds and equipped with feeders and water throughout the trial period, which consisted of 69 days, 15 days of adaptation of the animals to the facilities and diets and 54 days of data collection. At this stage, the animals received sorghum silage as roughage *ad libitum* and increasing proportions of experimental feeds. Goats were fed twice daily, with half the daily quota delivered at 8:00 and the remainder at 16:00. Feed portions were individually weighed in a roughage:concentrate ratio of 60:40 and subsequently mixed to minimize selective feeding by the animals.

Sorghum silage [*Sorghum bicolor* (L). Moench] was used as the roughage (Table 2). The concentrate was composed of cornmeal, soybean meal, a mineral supplement specific for goats, and crude glycerin. Diets were formulated to be isonitrogenous (150 g/kg of crude protein), according to the recommendations of the National Research Council (16), to meet nutritional requirements for goats with estimated potential average weight gains of 150 g/day.

**Table 2:** Chemical composition of ingredients used in the experimental diets.

Item	Ingredient			
	Sorghum silage	Corn grounded	Soybean meal	Crude glycerin
Dry matter	33.55	88.60	87.25	94.00
Organic matter^1^	96.71	98.46	93.52	96.40
Mineral matter^1^	3.29	1.54	6.48	3.60
Crude protein^1^	7.55	6.42	45.03	0.00
Ether extract^1^	3.05	5.15	1.84	0.00
Neutral detergent fiber^1^	49.03	13.07	15.46	0.00
Acid detergent fiber^1^	26.16	1.30	3.63	0.00
Nonfiber carbohydrate^1^	57.90	73.82	31.19	83.01
Total digestible nutrients^1^	55.00	81.07	80.11	81.30
Glycerol	0.00	0.00	0.00	43.4
Methanol	0.00	0.00	0.00	6.6

^1^Value expressed in % dry matter.

### 2.2. Metabolic profile and chemical composition of ingredients

To allow examination of the influence of crude glycerin levels on metabolic protein, energy, and enzymatic hepatic profiles, blood samples (10 mL) from each goat were collected from the jugular vein at the beginning, the middle and the end of the experimental period. The samples were collected before morning feeding (time 0) and 4 h thereafter in nonheparinized vacutainer tubes after local antisepsis. Samples were centrifuged at 3,500 rpm for 15 min to obtain the blood serum, which was transferred to duly labeled Eppendorf tubes and stored in a freezer at −20°C for subsequent analysis.

Samples were taken to the Laboratory of Clinical Pathology of the Federal Rural University of Pernambuco, where the serum concentrations of urea, total protein, albumin, creatinine, cholesterol, triglycerides, glucose, fructosamine, gamma-glutamyltransferase (GGT), alanine aminotransferase (ALT), aspartate aminotransferase (AST), and creatine kinase MB isoenzyme (CK-MB) were analyzed and quantified in the automatic biochemical analyzer LAB MAX 240 with commercial reagent kits from LABTEST^®^ (17, 18).

Urine samples from each goat were collected at the beginning, the middle and the end of the experimental period. The samples were collected before morning feeding (time 0) and 4 h thereafter on the 68th day of the experimental period, urine samples were collected from the animals at approximately 4 h after the morning feeding. Urine samples were collected in plastic cups from spontaneous urination, filtered with gauze, and a 10 mL aliquot was collected. Subsequently, samples were diluted in 40 mL of 0.036 N sulfuric acid solution (17, 18). Samples were then stored in labeled plastic containers at −20°C.

Subsequently, samples were sent to the Laboratory of Clinical Pathology, where urinary concentrations of urea, creatinine, urinary proteins, and uric acid were analyzed and quantified in the automatic biochemical analyzer LAB MAX 240 with commercial reagent kits from LABTEST^®^.

Animals were fasted on the last day of the experimental period; then, they were transferred to a commercial slaughterhouse in the municipality of Feira de Santana, state of Bahia, Brazil, where they were slaughtered in compliance with current regulations of Normative Instruction No. 3 of the Brazilian Ministry of Agriculture and Supply – Secretariat of Agricultural and Livestock Defense (9). Animals were slaughtered after being desensitized by electronarcosis and exsanguinated by severing the jugular vein and carotid artery. After skinning and evisceration of the animal, liver fragments of approximately 1 cm^2^ were sampled from each goat and transferred to individual containers with 10% buffered formalin.

Subsequently, the samples were taken to the Laboratory of Histology of the Animal Science Graduate Program of the Federal University of Paraíba, where they were initially processed by inclusion in paraffin. The analyzed area of histological sections was standardized at 4 μm, and sections were subsequently stained with hematoxylin and eosin (HE) for histopathological examination and evaluation of the effect of crude glycerin on liver tissues following (19). Morphological variables were microscopically evaluated and included hepatocyte swelling, parenchymal disruption, inflammatory infiltrate, steatosis, and congestion.

### 2.3. Statistical analysis

The data were subjected to analysis of variance in a completely randomized design with four treatments, named 0, 50, 100, and 150 g/kg inclusion of crude glycerin, and eight repetitions; the initial weight of the goats was considered as a covariate in the statistical model.

The results were interpreted through decomposition of the orthogonal polynomials in linear and quadratic using the PROC MIXED function of the SAS software (version 9.1). The homogeneity of variance between treatments was assumed, and the degrees of freedom were estimated using the Kenward-Roger method. The regression models were adjusted according to the significance of the parameters β1 and β2 by using the method of restricted maximum likelihood in PROC MIXED, and the estimation of parameters was obtained through the PROC REG function of the SAS software (version 9.1). All statistical procedures were performed using the value of 0.05 as the critical level of probability for error type I.

## 3. Results

### 3.1. Urea, total protein, albumin, and creatinine serum levels of goat kids

The diets did not have an effect (*p* > 0.05) on the serum urea levels (Table 3). Total serum protein concentrations were not affected (*p* > 0.05) in any samples from any examined diets.

**Table 3:** Urea, total protein (TP), albumin, and creatinine serum levels of goat kids fed diets containing crude glycerin.

Variable/h		Crude glycerin inclusion level (%)				SEM^1^	*p* value*	
		0	5	10	15		L^2^	Q^3^
Urea (mg/dL)	0 h	74.85	79.15	73.43	82.55	2.2712	0.3242	0.5393
	4 h	65.87	76.55	72.81	79.28	1.6554	0.4088	0.6274
TP (g/dL)	0 h	6.33	6.61	6.04	6.74	0.1131	0.4114	0.3989
	4 h	6.67	6.83	6.24	6.67	0.1120	0.1217	0.9665
Albumin (g/dL)	0 h	2.50	2.51	2.33	2.47	0.0385	0.8107	0.6808
	4 h	2.58	2.60	2.44	2.46	0.0377	0.8611	0.4933
Creatinine (mg/dL)	0 h	0.38	0.41	0.37	0.37	0.0181	0.4744	0.3057
	4 h	0.35	0.42	0.37	0.38	0.0194	0.4880	0.4893

^1^SEM, Standard error of the mean. ^2^Significance for linear effect. ^3^Significance for quadratic effect. 0 h = before morning feeding. 4 h = 4 h after morning feeding. mg/dL = milligrams per deciliter. g/dL = grams per deciliter. *p* value * = significant probability at the 5% level.

Serum albumin levels were not affected (*p* > 0.05) by the diets (Table 3). Serum creatinine concentrations were not affected (*p* > 0.05) by the diets, showing a mean of 0.38 mg/dL for both sampling times.

### 3.2. Energy profile of goat kids fed diets

Serum concentrations of plasma cholesterol and triglycerides at 0 h were not influenced (*p* > 0.05) by the diets; only triglycerides were influenced by the inclusion of crude glycerin (Table 4), and these decreased as the dietary glycerin doses increased.

**Table 4:** Energy profile of goat kids fed diets containing crude glycerin.

Metabolite		Crude glycerin inclusion level (%)				SEM^1^	*p* value*	
		0	5	10	15		L^2^	Q^3^
Cholesterol (mg/dL)	0 h	43.11	39.91	37.94	36.13	1.4156	0.2250	0.8485
	4 h	40.40	37.66	35.85	33.92	1.4467	0.2338	0.9191
Triglycerides (mg/dL)	0 h	13.07	11.12	12.12	11.74	0.6624	0.7743	0.7369
	4 h	18.55	16.47	16.03	15.53	1.3009	0.0433	0.7242
Glucose (mg/dL)	0 h	40.50	43.17	36.87	39.70	1.0684	0.3616	0.9699
	4 h	47.33	51.42	43.86	44.91	1.2726	0.1065	0.4524
Fructosamine (μmol/L)	0 h	191.68	196.75	189.51	196.61	2.2400	0.7131	0.8259
	4 h	202.19	207.88	194.09	199.96	2.3542	0.3006	0.9847
Triglycerides	4 h	Ŷ = −0.9529X + 19.0270				(R^2^ = 85.57)		

^1^SEM, Standard error of the mean. ^2^Significance for linear effect. ^3^Significance for quadratic effect. 0 h = before morning feeding. 4 h = 4 h after morning feeding. mg/dL = milligrams per deciliter. mg/dL = milligrams per deciliter. Μmol/L = micromole per liter. *p* value* = significant probability at the 5% level.

Serum concentrations of plasma glucose and fructosamine were not influenced (*p* > 0.05) by the diets (Table 4). Serum fructosamine values varied (*p* > 0.05) from 189.51 to 207.88 μmol/L.

### 3.3. Enzymatic activities of goat kids fed diets

Serum gamma-glutamyltransferase (GGT), alanine aminotransferase (ALT), and aspartate aminotransferase (AST) were not affected (*p* > 0.05) by the diets (Table 5).

**Table 5:** Enzymatic activities of gamma-glutamyltransferase (GGT), alanine aminotransferase (ALT), aspartate aminotransferase (AST), and creatine kinase myocardial isoenzyme (CK-MB) in Boer crossbred goat kids fed diets containing crude glycerin.

Variable/h		Crude glycerin inclusion level (%)				SEM^1^	*p* value*	
		0	5	10	15		L^2^	Q^3^
GGT (UI/L)	0 h	46.96	47.97	46.59	53.76	1.3257	0.1767	0.1967
	4 h	49.41	50.39	51.52	51.45	1.0063	0.4747	0.8163
AST (UI/L)	0 h	53.27	49.98	43.56	51.83	2.3206	0.5634	0.1678
	4 h	63.68	60.55	52.19	61.24	1.9290	0.3788	0.1295
CK-MB (UI/L)	0 h	190.68	189.21	193.69	192.00	2.2218	0.7029	0.9822
	4 h	194.13	193.79	186.57	192.23	2.2261	0.5414	0.5267

^1^SEM = Standard error of the mean. ^2^Significance for linear effect. ^3^Significance for quadratic effect. 0 h = before morning feeding. 4 h = 4 h after morning feeding. mg/dL = milligrams per deciliter. g/dL = grams per deciliter. *p* value * = significant probability at the 5% level.

Serum activity of the creatine kinase myocardial isoenzyme (CK-MB) was not affected (*p* > 0.05) by the diets (Table 5), showing a mean value of 191.54 IU/L.

### 3.4. Histopathological lesions of the hepatic tissue of goat kids fed diets

The liver of animals receiving the treatment without added crude glycerin showed normal architecture without histopathological changes (Table 6), except for one animal that showed cell swelling and congestion but not enough to characterize such histological alteration. Animals receiving 50 g/kg crude glycerin showed similar characteristics to those fed without glycerin, with two animals showing early cell (hepatocyte) swelling insufficient to characterize this alteration, one showing moderate microvacuolar steatosis, and one showing congestion (Table 6).

**Table 6:** Histopathological lesions of the hepatic tissue of Boer x undefined breed (UDB) goats fed diets containing crude glycerin.

Histopathological lesion (%)	Crude glycerin inclusion level (%)			
	0	5	10	15
Hepatocyte swelling	0.00	0.00	0.00	37.50
Parenchymal disruption	0.00	0.00	12.50	37.50
Inflammatory infiltrate	0.00	0.00	25.00	0.00
Steatosis	0.00	12.50	50.00	37.50
Congestion	0.00	12.50	12.50	37.50

**Figure 1:**
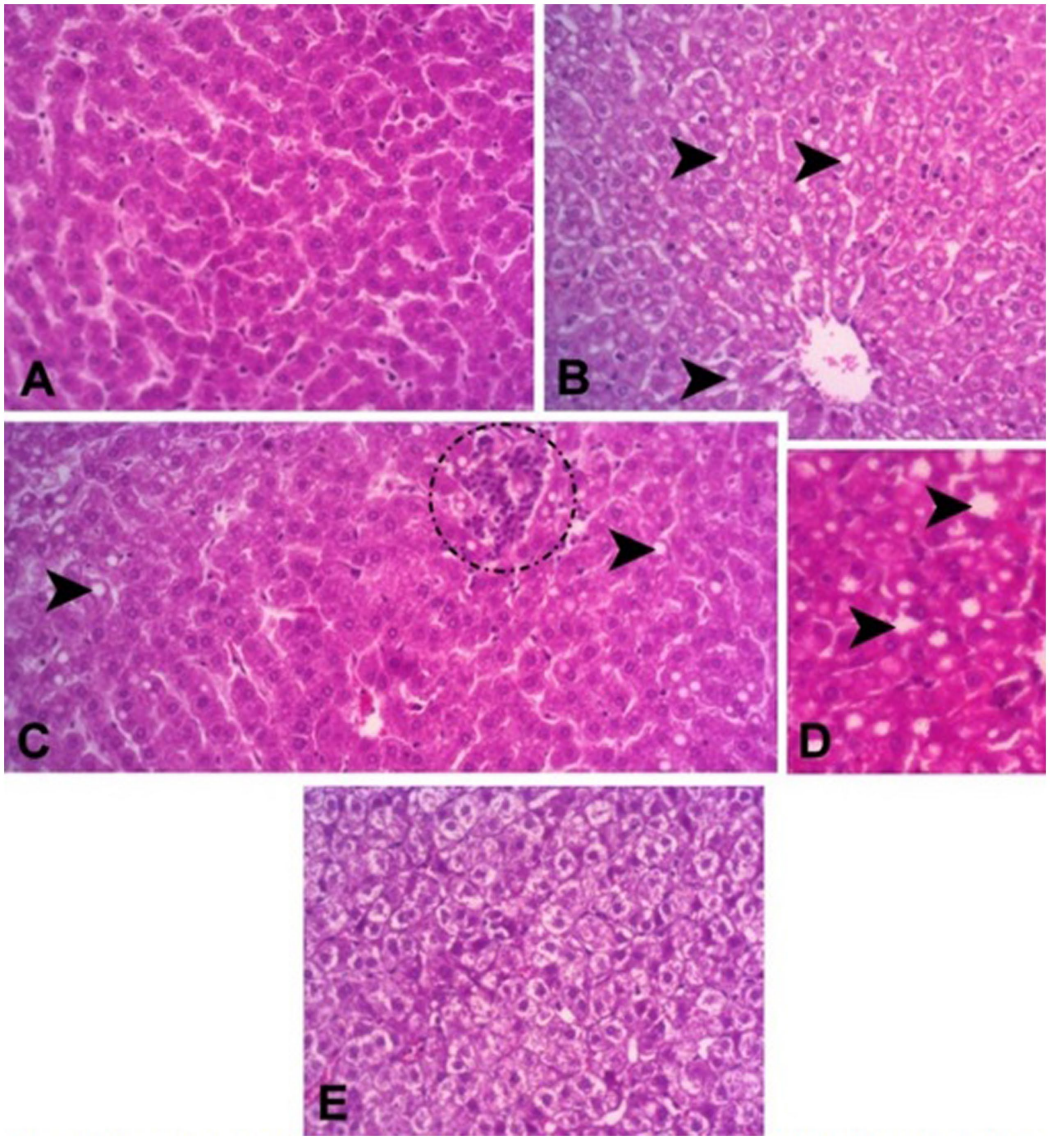
Liver photomicrographs showing histopathological changes in Boer crossbred goats fed diets containing crude glycerin. **(A)** Normal hepatic parenchyma showing normal architecture, representing the liver of animals from treatment 1. **(B)** Parenchyma with moderate microvacuolar steatosis, representing mainly animals from treatments 3 and 4. **(C)** Hepatic parenchyma of an animal from treatment 3, showing moderate steatosis and inflammatory infiltrate (dashed circle). **(D)** Increased steatotic hepatic parenchyma of an animal from treatment 4. **(E)** Representative parenchyma of animals from treatment 4, showing parenchymal disruption and hepatocyte swelling. The arrowhead indicates lipid vacuoles indicative of steatosis. Hematoxylin–eosin stained.

Hepatic histopathological changes were more frequent in the treatment with 100 g/kg crude glycerin compared to the first two treatments. Liver slides showed parenchymal disruption, inflammatory infiltrate, moderate microvacuolar steatosis, and hepatic congestion (Table 6; Figure 1). Steatosis values were particularly noteworthy, with approximately 50% of the animals in this treatment showing the condition. All histopathological changes examined in this study were found in the treatment with the highest dose of crude glycerin, except inflammatory infiltrate (Table 6). Among the cases of steatosis, one was severe.

## 4. Discussion

### 4.1. Urea, total protein, albumin, and creatinine serum levels of goat kids

The serum urea levels (Table 3) slightly exceeded the limits of normality described for goats. According to Kaneko et al. (17), standard values between 21.4 and 42.8 mg/dL. Nichols et al. (20) states that the serum urea levels exceeding the normal range are caused by excessive protein intake, low energy intake, or even asynchronous degradation of energy and protein, the latter of which might explain the values found in this study. Although the diets and protein and energy intakes were similar among the treatments (because diets were isoproteic and isoenergetic), the treatments with crude glycerin showed the highest serum urea values; therefore, the availability of glycerol from glycerin may have aggravated urea levels because glycerol is energy-rich and rapidly absorbed by the ruminal epithelium, whereas the main source of dietary protein (soybean meal) is not degraded as rapidly.

The mean total serum protein value found in this study was 6.52 g/dL, which lies in the range of 6.4–7.0 g/dL recommended by Kaneko et al. (17). According to Nichols et al. (20), reduced protein levels may be associated with blood loss or nutritional deficiencies that promote organic impairment. These findings indicate that the animals did not have any nutritional deficiencies.

The serum albumin levels approach the values of normality recommended for the species (17). Regarding serum creatinine concentrations, the reference values for goats reported by Kerr (18) showed a variation of 1.0–1.8 mg/dL. All treatments employed in this study resulted in creatinine values below the lower limit. Creatinine is almost entirely derived from the catabolism of the creatine present during muscle metabolism and reflects the renal filtration rate, meaning that high creatinine levels are indicative of altered renal functions (21, 22).

### 4.2. Energy profile of goat kids fed diets

The triglycerides decreased as the dietary glycerin doses increased. This may be due to a decreased ether extract intake, given that the ether extract content was reduced by the inclusion of the crude glycerin in the diets (Table 1). However, values of all diets were below the normal range compared to other studies with goats (22–24). Notwithstanding, the results found in this study are desirable because the current consumer market has been seeking low-fat food products, and the lower contents found in the blood plasma of the goats in this study are good indicators that little fat deposition occurred in the muscle tissue.

Mean glucose values were at 36.87–51.42 mg/dL, slightly below the reference values of 50–75 mg/dL cited by Kaneko and Kerr (17, 18). Glucose can be used as a parameter to assess metabolic energy-although results from the monitoring of ruminant energy metabolism are inconsistent-and is usually measured by acetic, propionic, and butyric short-chain fatty acids. Hence, the fact that the glucose values found by this study were slightly below those recommended by Kaneko (17) is not concerning.

Reference values highlighting the importance of fructosamine blood concentrations in ruminants are still understudied in Brazil. In a comparison of sample collection times in sheep and goats, Kuru et al. (21) found blood fructosamine values ranging from 164.68 to 328.88 μmol/L for small ruminants. Those values can be used as a reference, but more studies are warranted to establish reference values when considering variation in different factors. As such, the values found in the present study are within the range observed by Kuru et al. (21).

### 4.3. Enzymatic activities of goat kids fed diets

The values of serum gamma-glutamyltransferase (GGT), alanine aminotransferase (ALT), and aspartate aminotransferase (AST) remained within the normal range for the species according to Kaneko et al. (17), who preconized normal AST values of 20–56 IU/L and GGT values of 20–70 IU/L. The mean values observed in this study were 53.98 IU/L and 49.76 IU/L for AST and GGT, respectively, indicating that hepatic function was not compromised. According to Bobe et al. (25), the increase in the enzymatic serum rates from the liver tissue is related to hepatocellular disease, given that the degree of increase is directly proportional to the number of affected hepatocytes.

The creatine kinase myocardial isoenzyme (CK-MB) is an isoenzyme found mainly in the heart and whose quantification can be applied reliably in diagnosing acute myocardial infarction in humans. The enzyme has been well-studied in rodents (26, 27) but is not entirely reliable in field evaluations because of its short half-life (28). Notwithstanding, Pedroso (29) found altered CK-MB values in cattle intoxicated by *Nerium oleander*, most commonly known as oleander, whose ingestion by animals leads to several clinical symptoms such as arrhythmia, paralysis, and even death. The authors also observed an effect, where values ranged from 158–206 IU/L to 402–285 IU/L when animals were intoxicated with 0.5 and 1.0 g/kg body weight, and noted areas of extensive hyaline necrosis in the heart papillary muscle. Such findings demonstrate CK-MB also might be indicative of cardiac problems in ruminants.

### 4.4. Histopathological lesions of the hepatic tissue of goat kids fed diets

The different individual responses of the study animals to these characteristics may indicate hepatic overload, as goat kids fed the highest levels of crude glycerin were more often affected by histopathological lesions and with greater occurrence, which was not verified (Table 6). This hepatic overload suffered by animals fed higher glycerin levels may be related to the excess glycerol that is readily absorbed by the ruminal epithelium, metabolized in the liver, and directed to gluconeogenesis, or else by the methanol also present in the glycerin.

This response demonstrates that crude glycerin inclusion levels did not lead to any kidney problems in goats, thus reducing the risk of being affected by diseases such as urolithiasis, one of the main diseases that affects the urinary tract of ruminants, and avoiding economic losses related to veterinary treatment expenses, death of affected animals, and carcass condemnation at emergency slaughter.

The different diets did not affect the metabolic, protein, or energy profile of the goats examined, with the profiles being within normal limits for the species for most of the evaluated parameters. Although the findings from the histopathological examination of the liver tissue indicates moderate overload, the diets did not significantly affect the body of the study animals. Therefore, during the evaluated period and in the crude glycerin levels studied, this co-product can be recommended as an energy source for the diet of goat kids, given that deleterious effects were not observed.

The indicate crude glycerin did not cause deleterious effects on renal tissue or on the protein, energy, or enzymatic serum profiles and resulted in only moderate damage to the liver tissue.

## Data availability statement

The raw data supporting the conclusions of this article will be made available by the authors, without undue reservation.

## Ethics statement

The animal study was approved by Federal University of Bahia Animal Use and Care Committee (n. 08/2013). The study was conducted in accordance with the local legislation and institutional requirements.

## Author contributions

HB, ES, GC, and JO: conceptualization. GC and JO: data curation. HB and ES: formal analysis. ES and JO: funding acquisition and supervision. HB and MC: investigation. HB, JO, and MC: methodology. ES: project administration. RG, DP, DF, TN, and AZ: resources. FS: software. FS, RG, and DP: validation. HB, ES, GC, JO, FS, MC, RG, DP, DF, TN, and AZ: visualization. HB, ES, and FS: writing – original draft. DP, DF, TN, and AZ: writing – review and editing. All authors contributed to the article and approved the submitted version.

## Funding

This study was supported by Coordination for the Improvement of Higher Education Personnel (CAPES) (Finance code 001), FAPEMA (Maranhão State Research Foundation) and FAPESB (Bahia State Research Foundation) for its financial support.

## Conflict of interest

The authors declare that the research was conducted in the absence of any commercial or financial relationships that could be construed as a potential conflict of interest.

## Publisher’s note

All claims expressed in this article are solely those of the authors and do not necessarily represent those of their affiliated organizations, or those of the publisher, the editors and the reviewers. Any product that may be evaluated in this article, or claim that may be made by its manufacturer, is not guaranteed or endorsed by the publisher.
